# Supplementary material for: Immunomodulatory Activities of Carica papaya L. Leaf Juice in a Non-Lethal, Symptomatic Dengue Mouse Model
Source: Pathogens. 2021 Apr 21;10(5):501. doi: 10.3390/pathogens10050501 (PMC8170887; doi:10.3390/pathogens10050501)
Supplement: Supplementary file 1 [file pathogens-10-00501-s001.zip › pathogens-1147596-SI.pdf]

Table S1. HRMS/MS description of the reference standards

Standard	RT (min)	Molecular formula	Monoisotopic mass	*m/z*	Mass error (ppm)	MS/MS fragments (Top 5)
Manghaslin	4.94	$C_{33}H_{40}O_{20}$	756.2112	755.2036 $[M-H]^-$	0.62	255.03 (4.03), 271.02(6.46), 284.03 (9.06), 300.03 (100), 301.03 (20.43)
Clitorin	5.10	$C_{33}H_{40}O_{19}$	740.2163	739.2083 $[M-H]^-$	0.99	227.04 (2.64), 255.03 (8.55), 256.04 (3.16), 284.03 (100), 285.04 (24.78)
Rutin	5.23	$C_{27}H_{30}O_{16}$	610.1533	609.1452 $[M-H]^-$	1.49	151.00 (5.65), 178.99 (6.91), 300.03 (100), 301.03 (100), 302.04 (7.74)
Nicotiflorin	5.43	$C_{27}H_{30}O_{15}$	594.1580	593.1501 $[M-H]^-$	1.76	255.03 (6.90), 257.05 (3.14), 284.03 (72.33), 285.04 (100), 286.04 (16.21)
Carpaine	5.58	$C_{28}H_{50}N_2O_4$	478.3770	479.3836 $[M+H]^+$	0.75	222.18 (9.49), 240.20 (100), 241.20 (16.35), 479.38 (7.94), 480.39 (3.21)

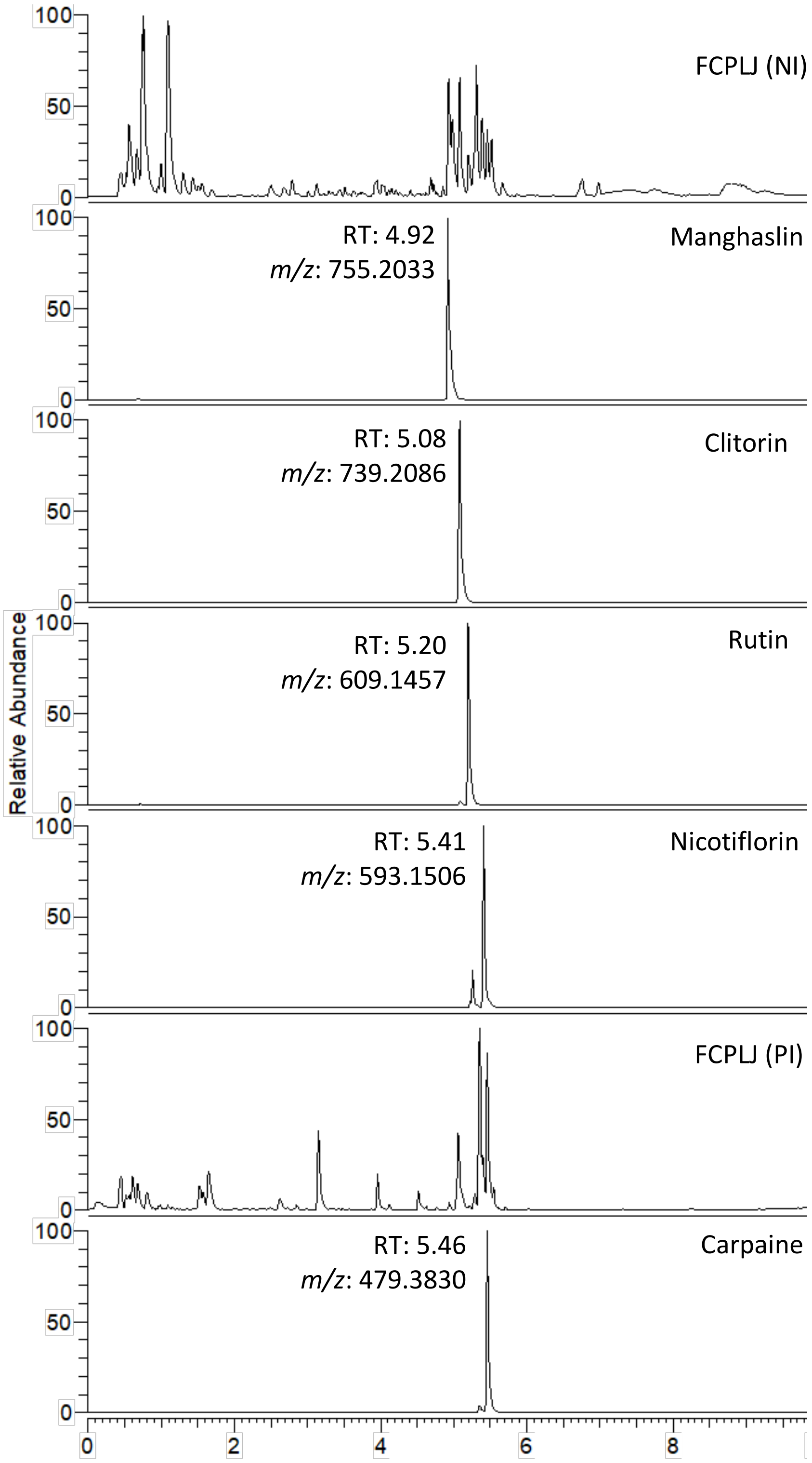

Figure S1. Base peak chromatogram for 5 mg/mL of FCPLJ and the extracted ion chromatograms (5 ppm) of five chemical markers. Manghaslin, clitorin, rutin and nicotiflorin were acquired in negative ionization (NI) and carpaine was acquired in positive ionization (PI). Retention time (RT) are presented in minute.

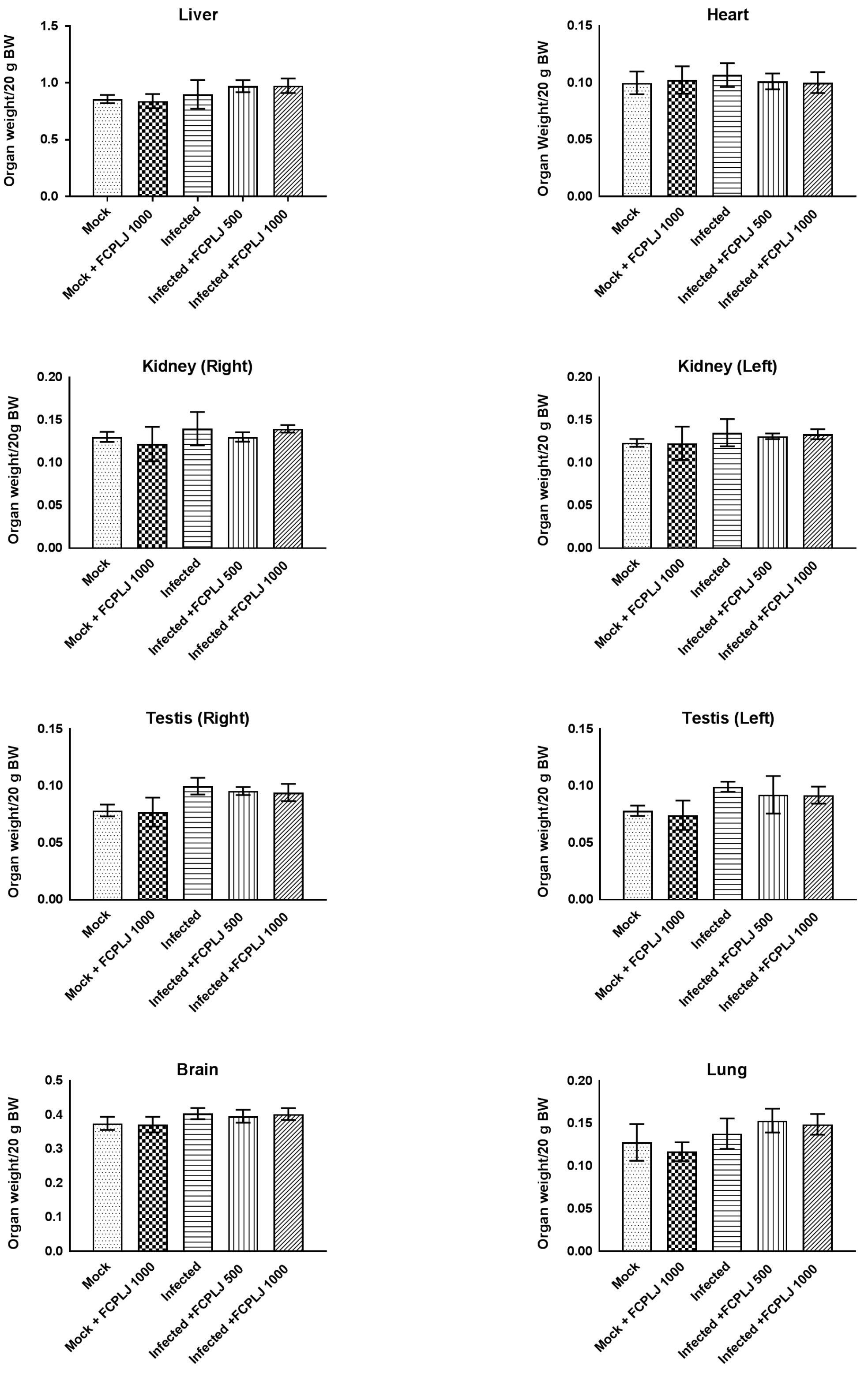

Figure S2. The organs weight of AG129 mice infected with dengue virus. The organs were harvested on day 4 post infection and the weight of each organs was normalized against 20g bodyweight. The bars represent the mean values ± standard deviation. The comparisons between groups were analyzed by ANOVA using Tukey's multiple comparison test. Each of experimental group consist of 5 mice (n=5).

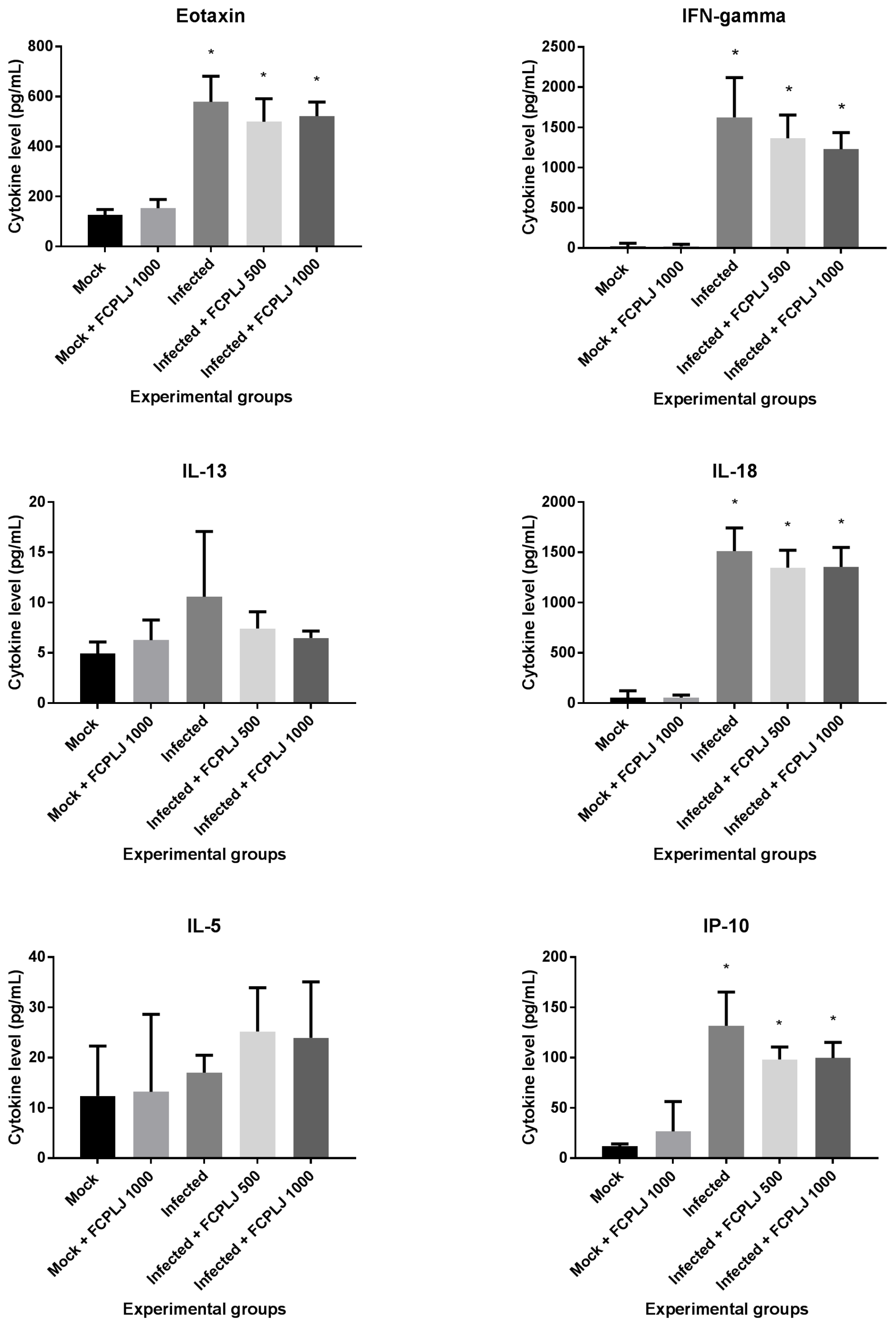

Figure S3. The cytokines level in the plasma of experimental AG129 mice groups. The cytokine levels in the plasma were detected by multiplex assay system. The bars represent the mean values ± standard deviation. The comparisons between groups were analyzed by ANOVA using Tukey's multiple comparison test. Each of experimental group consist of 5 mice (n=5). The asterisk (*) represents significant difference ($p<0.05$) when compared with mock infected and mock + FCPLJ 1000 mice.

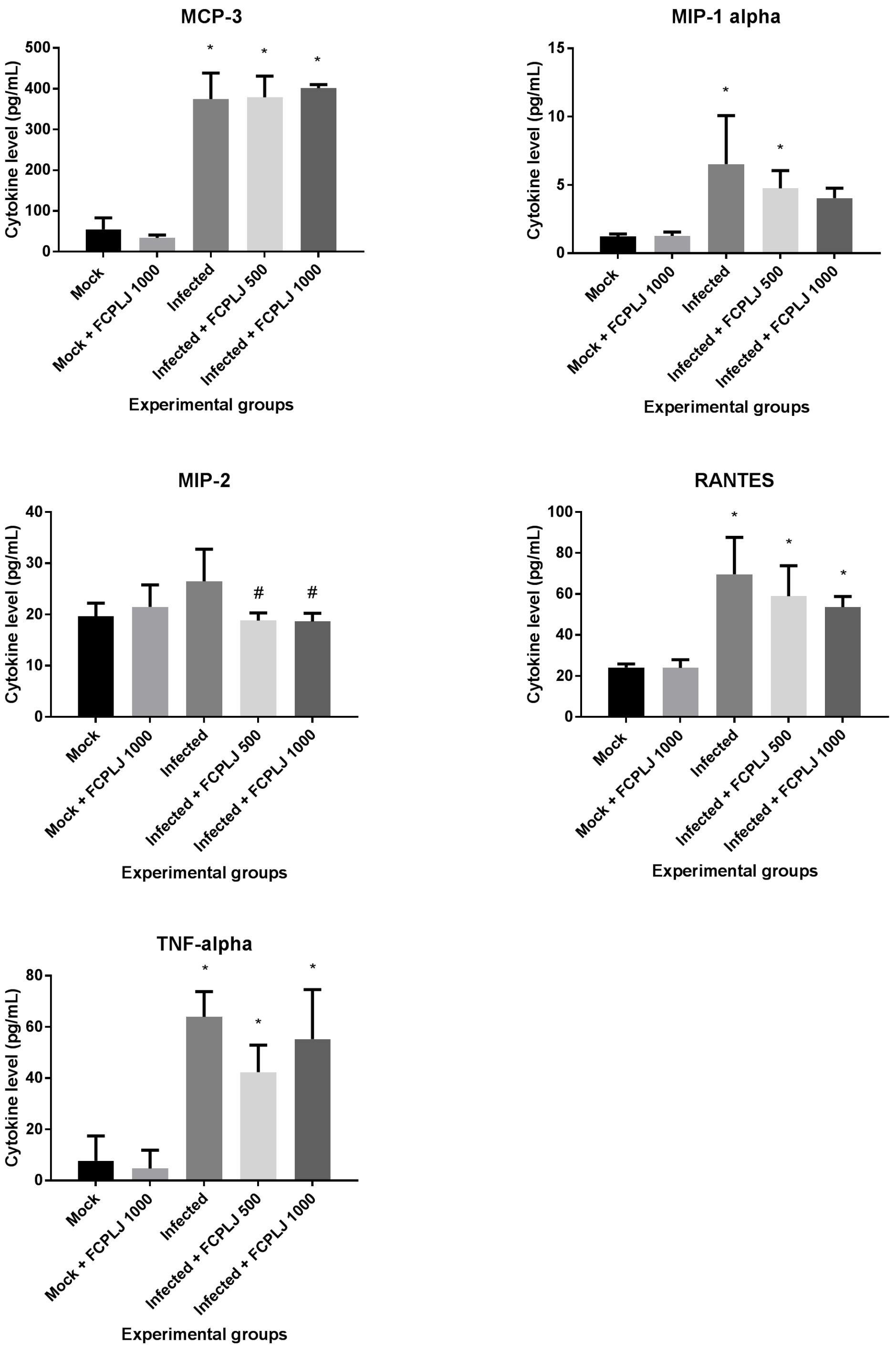

Figure S3 (continued). The cytokines level in the plasma of experimental AG129 mice groups. The cytokine levels in the plasma were detected by multiplex assay system. The bars represent the mean values ± standard deviation. The comparisons between groups were analyzed by ANOVA using Tukey's multiple comparison test. Each of experimental group consist of 5 mice (n=5). The asterisk (*) represents significant difference ($p<0.05$) when compared with mock infected and mock + FCPLJ 1000 mice.

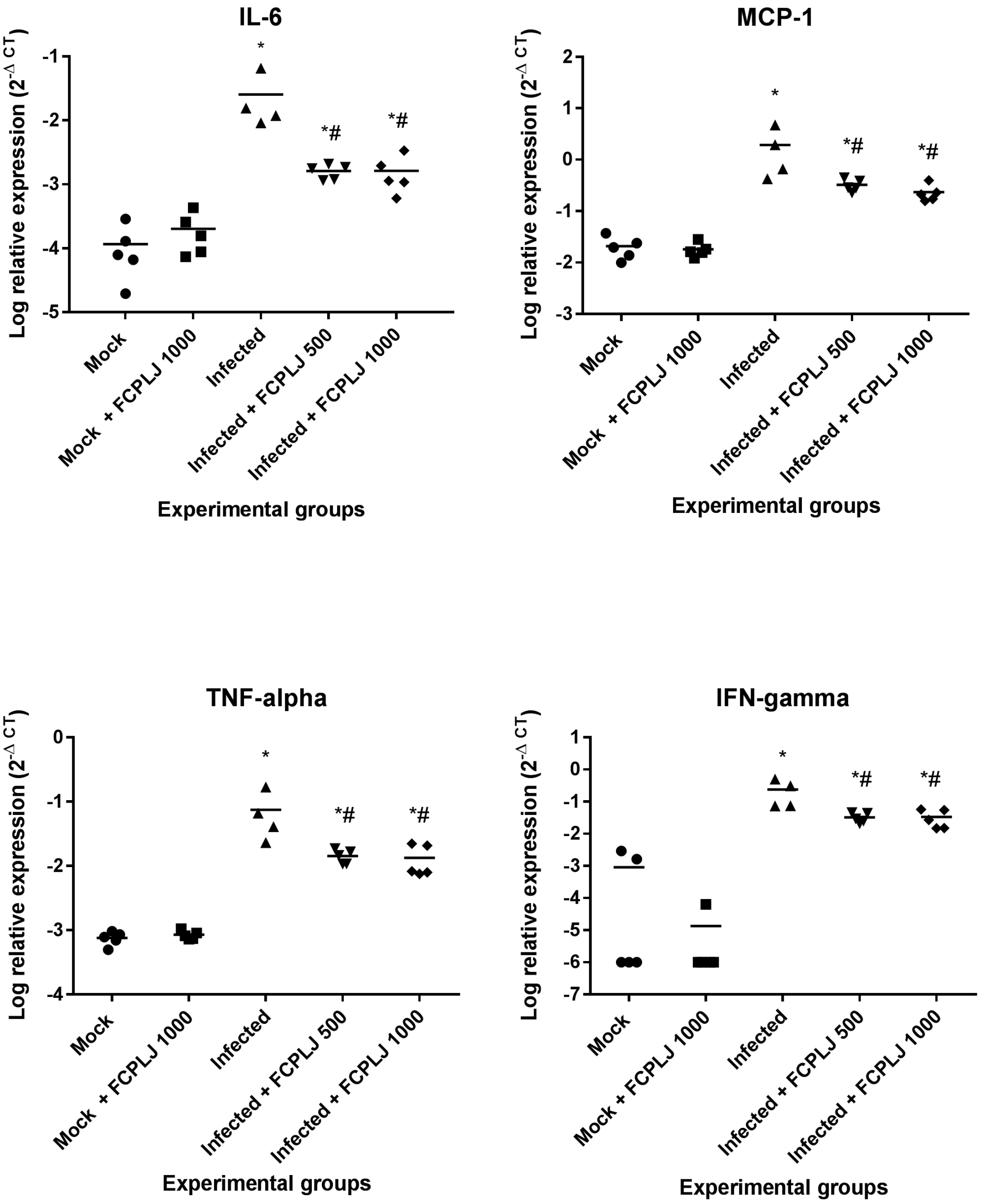

Figure S4. Intracellular cytokine expression levels in kidney tissues. The expression level of four cytokines (MCP-1, IL6, IFN and TNF) in the kidney tissues were determined by quantitative reverse transcription PCR. The comparisons between groups were analyzed by ANOVA using Tukey's multiple comparison test. Note: The asterisk (*) represents significant difference ($p < 0.05$) when compared with mock-infected and mock + FCPLJ 1000 mice. The hash (#) represents a significant difference ($p < 0.05$) when compared with the infected mice. Each of experimental group consist of 5 mice (n=4-5).

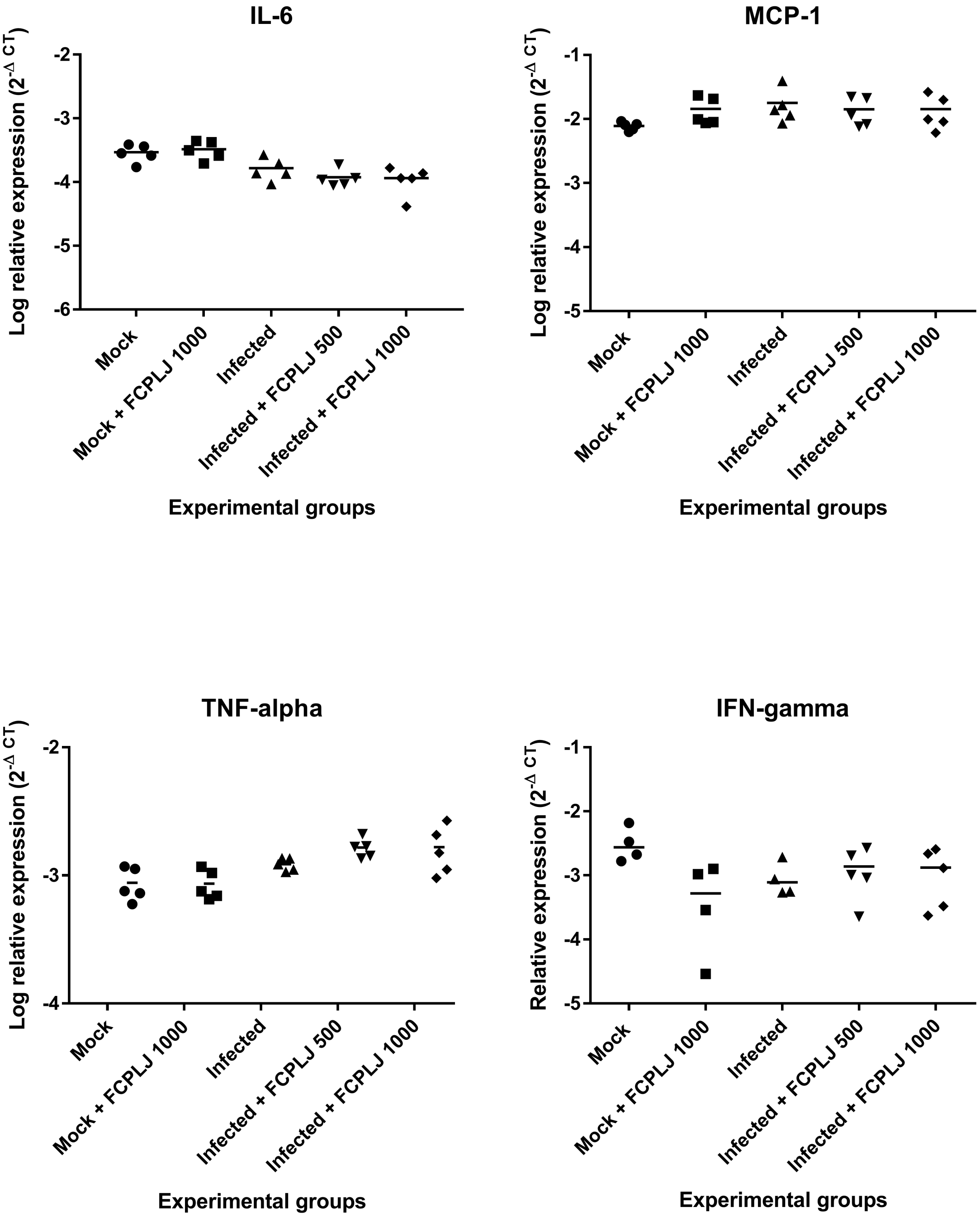

Figure S4 (continued). Intracellular cytokine expression levels in brain tissues. The expression level of four cytokines (MCP-1, IL6, IFN and TNF) in the brain tissues were determined by quantitative reverse transcription PCR. The comparisons between groups were analyzed by ANOVA using Tukey's multiple comparison test. Note: The asterisk (*) represents significant difference ($p<0.05$) when compared with mock infected and mock + FCPLJ 1000 mice. Each of experimental group consist of 5 mice (n=5).

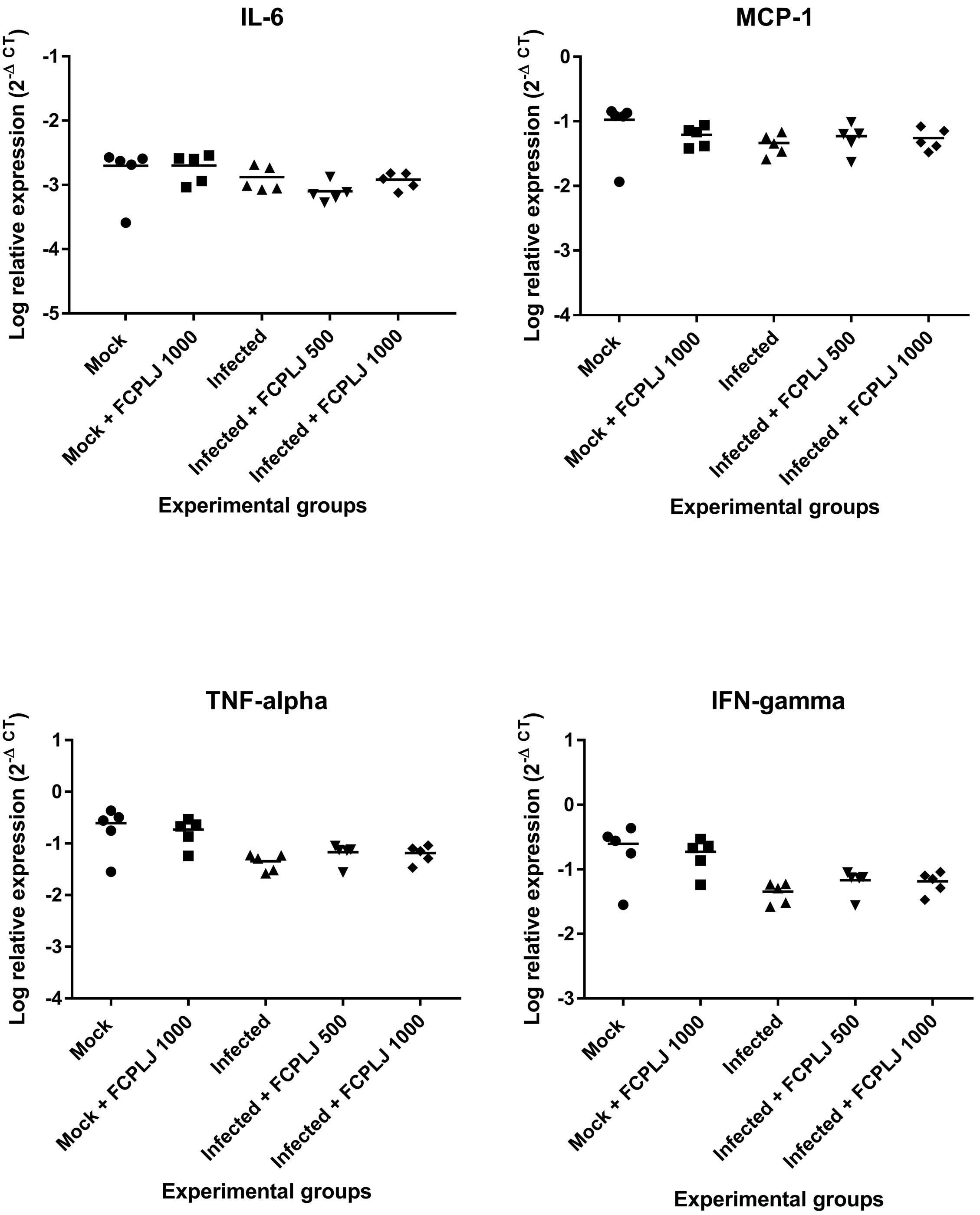

Figure S4 (continued). Intracellular cytokine expression levels in spleen tissues. The expression level of four cytokines (MCP-1, IL6, IFN and TNF) in the spleen tissues were determined by quantitative reverse transcription PCR. The comparisons between groups were analyzed by ANOVA using Tukey's multiple comparison test. Note: The asterisk (*) represents significant difference ($p<0.05$) when compared with mock infected and mock + FCPLJ 1000 mice. Each of experimental group consist of 5 mice (n=5).

Table S2. The functions of cytokines affected by *C. papaya* leaf juice treatment in AG129 mice infected with clinical DENV-2 isolate.

No	Cytokine	Cytokine description	UniProt ID[a]	General function[a]	Function in dengue
**1**	GM-CSF	Granulocyte-macrophage colony-stimulating factor	P01587	Stimulates the growth and differentiation of hematopoietic precursor cells from various lineages, including granulocytes, macrophages, eosinophils and erythrocytes.	Produced by human skin fibroblast after dengue virus infection (1). Function in activation of proinflammatory macrophages during dengue infections. Activated macrophages secreted the IL1-β, IL-18 through CLEC5A-NLRP3 inflammasome pathway (2).
**2**	GRO-α (CXCL1)	growth-regulated protein alpha	P12850	Has chemotactic activity for neutrophils. May play a role in inflammation and exerts its effects on endothelial cells in an autocrine fashion.	Expression increased in DEN-2 infected macrophage *in vitro* (3). Dengue patients with plasma leakage showed decrease in GRO-α serum level (4).
**3**	IL-1β	Interleukin-1 beta	P10749	Potent proinflammatory cytokine. Initially discovered as the major endogenous pyrogen, induces prostaglandin synthesis, neutrophil influx and activation, T-cell activation and cytokine production, B-cell activation and antibody production, and fibroblast proliferation and collagen production.	Expression of IL-1β increased via caspase 4 activation in DEN-2 infected macrophage *in vitro* (5). Exposure of platelet from dengue patients to monocytes from healthy volunteers induced the IL-1β and other cytokines, IL-8, IL-10 and MCP-1 (6). IL-1β was secreted from platelet infected with dengue virus. Platelet-derived IL-1β contribute to increased vascular permeability in dengue infection (7).
**4**	IL-6	Interleukin-6	P08505	Cytokine with a wide variety of biological functions. It is a potent inducer of the acute phase response. Plays an essential role in the final differentiation of B-cells into Ig-secreting cells Involved in lymphocyte and monocyte differentiation. Acts on B-cells, T-cells, hepatocytes,	Increased in dengue virus-infected monocyte culture (8). Serum level of IL-6 was high in dengue patients with plasma leakage (4).

				hematopoietic progenitor cells and cells of the CNS. Required for the generation of T(H)17 cells. Also acts as a myokine. It is discharged into the bloodstream after muscle contraction and acts to increase the breakdown of fats and to improve insulin resistance. It induces myeloma and plasmacytoma growth and induces nerve cells differentiation.	
**5**	MCP-1 (CCL2)	Monocyte chemoattractant protein 1	P10148	Chemotactic factor that attracts monocytes and basophils but not neutrophils or eosinophils. Augments monocyte anti-tumor activity. Has been implicated in the pathogenesis of diseases characterized by monocytic infiltrates, like psoriasis, rheumatoid arthritis or atherosclerosis. May be involved in the recruitment of monocytes into the arterial wall during the disease process of atherosclerosis.	Exposure of platelet from dengue patients to monocytes from healthy volunteers induced the MCP-1 and other cytokines, IL-8, IL-10 and IL-1β (6). The expression of MCP-1 was high in patients with dengue haemorrhagic fever (9).
**6**	MIP-1β (CCL4)	Macrophage inflammatory protein-1 beta	P14097	Monokine with inflammatory and chemokinetic properties. Binds to CCR5. One of the major HIV-suppressive factors produced by CD8+ T-cells. Recombinant MIP-1-beta induces a dose-dependent inhibition of	Increased MIP-1β and MCP-1 levels in dengue patients during acute infection (10). MIP-1β was found to be increased in patients with warning sign during febrile phase and patients without warning sign during defervescence (11).

		different strains of HIV-1, HIV-2, and simian immunodeficiency virus (SIV).	

Note: [a]The Universal Protein Resource (UniProt) database (www.uniprot.org).

References

1. Kurane I, Janus J, Ennis FA. Dengue virus infection of human skin fibroblasts in vitro production of IFN-beta, IL-6 and GM-CSF. Arch Virol. 1992;124(1-2):21-30.
2. Wu MF, Chen ST, Yang AH, Lin WW, Lin YL, Chen NJ, et al. CLEC5A is critical for dengue virus-induced inflammasome activation in human macrophages. Blood. 2013;121(1):95-106.
3. Moreno-Altamirano MM, Romano M, Legorreta-Herrera M, Sanchez-Garcia FJ, Colston MJ. Gene expression in human macrophages infected with dengue virus serotype-2. Scand J Immunol. 2004;60(6):631-8.
4. Tramontini Gomes de Sousa Cardozo F, Baimukanova G, Lanteri MC, Keating SM, Moraes Ferreira F, Heitman J, et al. Serum from dengue virus-infected patients with and without plasma leakage differentially affects endothelial cells barrier function in vitro. PLoS One. 2017;12(6):e0178820.
5. Cheung KT, Sze DM, Chan KH, Leung PH. Involvement of caspase-4 in IL-1 beta production and pyroptosis in human macrophages during dengue virus infection. Immunobiology. 2018;223(4-5):356-64.
6. Hottz ED, Medeiros-de-Moraes IM, Vieira-de-Abreu A, de Assis EF, Vals-de-Souza R, Castro-Faria-Neto HC, et al. Platelet activation and apoptosis modulate monocyte inflammatory responses in dengue. J Immunol. 2014;193(4):1864-72.
7. Hottz ED, Lopes JF, Freitas C, Valls-de-Souza R, Oliveira MF, Bozza MT, et al. Platelets mediate increased endothelium permeability in dengue through NLRP3-inflammasome activation. Blood. 2013;122(20):3405-14.
8. Levy A, Valero N, Espina LM, Anez G, Arias J, Mosquera J. Increment of interleukin 6, tumour necrosis factor alpha, nitric oxide, C-reactive protein and apoptosis in dengue. Trans R Soc Trop Med Hyg. 2010;104(1):16-23.
9. Lee YR, Liu MT, Lei HY, Liu CC, Wu JM, Tung YC, et al. MCP-1, a highly expressed chemokine in dengue haemorrhagic fever/dengue shock syndrome patients, may cause permeability change, possibly through reduced tight junctions of vascular endothelium cells. J Gen Virol. 2006;87(Pt 12):3623-30.
10. de-Oliveira-Pinto LM, Marinho CF, Povoa TF, de Azeredo EL, de Souza LA, Barbosa LD, et al. Regulation of inflammatory chemokine receptors on blood T cells associated to the circulating versus liver chemokines in dengue fever. PLoS One. 2012;7(7):e38527.
11. Rathakrishnan A, Wang SM, Hu Y, Khan AM, Ponnampalavanar S, Lum LC, et al. Cytokine expression profile of dengue patients at different phases of illness. PLoS One. 2012;7(12):e52215.
